# Beyond the Screen: Reframing Virtual Reality as an Accessibility Technology for Autistic Individuals Through the Social Model of Disability

**DOI:** 10.31083/AP56107

**Published:** 2026-10-16

**Authors:** Sergio Machado, Alberto Souza de Sá Filho, Rafael Ferreira-Garcia, Lucio Lage Gonçalves, Federica Sancassiani, Giulia Cossu, Alessandra Perra, Mauro Giovanni Carta, Henning Budde, José Carlos Appolinario, Antonio Egidio Nardi

**Affiliations:** ^1^Center for Neuroscience, Neurodiversity Institute, Nova Iguaçu, RJ 26210-270, Brazil; ^2^LABPR—Panic and Respiration Laboratory, Institute of Psychiatry (IPUB), Federal University of Rio de Janeiro (UFRJ), Rio de Janeiro, RJ 21941-908, Brazil; ^3^Virtual Reality in Mental Health Laboratory (RVSM), Institute of Psychiatry (IPUB), Federal University of Rio de Janeiro (UFRJ), Rio de Janeiro, RJ 21941-908, Brazil; ^4^Graduate Program, Evangelical University of Goiás (UniEVANGÉLICA), Anápolis, GO 75083-515, Brazil; ^5^Department of Medical Sciences and Public Health, University of Cagliari, 09124 Cagliari, Italy; ^6^Department of Pedagogy, Institute for Systems Medicine (ISM), MSH Medical School Hamburg, University of Applied Sciences and Medical University Hamburg, 20457 Hamburg, Germany; ^7^Obesity and Eating Disorders Group (GOTA), Institute of Psychiatry, Federal University of Rio de Janeiro, Rio de Janeiro, RJ 22290-140, Brazil

**Keywords:** autism spectrum disorder, virtual reality, social model of disability, neurodiversity, safety, environmental accessibility

## Abstract

Virtual Reality (VR) has been increasingly highlighted as a promising therapeutic tool for autistic individuals, with meta-analyses indicating significant benefits in social and emotional aspects. However, a substantial portion of this research has been conducted within a medical model of deficit correction, focused primarily on standardizing and normalizing autistic behavior. This article proposes a profound shift in this perspective, viewing VR as an environmental accessibility technology grounded in the Social Model of Disability. Supported by the principles of neurodiversity, we argue that the true utility of VR lies in reducing the barriers that generate disability, not in correcting neurological differences. A critical analysis of the safety literature examined here suggests that certain adverse effects appear to be overlooked or underreported in a substantial proportion of the studies reviewed. Symptoms such as cybersickness, sensory overload, anxiety, and post-session fatigue pose real and immediate risks when considering the sensory processing profiles of autistic individuals. Thus, to foster more ethical and sustainable practices, we propose four interconnected priorities: systematic safety research, participatory co-design led by the community itself, implementation science in real-world contexts, and outcome metrics that genuinely affirm neurodiversity. By redefining what constitutes success in an intervention, prioritizing environmental fit, autonomy, and quality of life over behavioral conformity, this shift aligns the development of immersive technologies with the lived experiences of the autistic community. In doing so, it transforms VR from a corrective mechanism into a tool for affirmation and ecological adaptation.

## Main Points

1. Much of the Virtual Reality (VR) research on autism still starts from the assumption that autistic behavior needs correcting, aiming to normalize people rather than accommodate a real neurological difference.

2. In the studies we reviewed, adverse reactions, from cybersickness and sensory overload to anxiety and fatigue, were rarely reported; when they were, they varied considerably with the person’s sensory profile and the hardware used, as a recent systematic review showed. That these effects so often go undocumented in the studies examined is itself a significant gap in the evidence.

3. The Social Model of Disability distinguishes between impairment and disability, reframing the use of VR as a form of environmental accessibility that reduces disabling barriers rather than attempting to erase individual differences.

4. A research agenda is needed that prioritizes safety, a co-creation process led by the autistic community, and neurodiversity-affirming outcomes, while also tracking participants over time in real-world contexts.

## 1. Introduction

In recent years, Virtual Reality (VR) has become a strong bet, almost a promise, in the treatment of autistic people. And observing the findings in the literature, at first glance, they are impressive. Recent meta-analyses have found significant effects of VR-based interventions on social and emotional skills, and cognitive functions, with impacts that seem particularly relevant in activities of daily living [1,2,3]. The logic behind this is attractive, as VR creates immersive and controllable environments where the individual can practice skills, face situations that normally generate anxiety, and develop competencies, all within a safe, repeatable, and predictable context [4,5]. However, this rapid pace of research has mostly occurred within a very specific theoretical framework, where VR is treated as a tool to remedy deficits. The framework certainly has its merits, as it has produced positive results. But it raises uncomfortable questions about the assumptions guiding the development of each new intervention. What exactly are we trying to address? And for whom, ultimately, is this technology being designed? What if VR’s effectiveness comes not so much from its ability to correct autistic functioning, but rather from its ability to modify environmental barriers that hinder participation?

Within this context, the Social Model of Disability is an alternative to the predominantly medical model. This model, which emerged from studies on disability [6,7], starts from a simple idea: disability is not in a person’s body; it is not a characteristic they carry as a closed diagnosis. It arises from the encounter between the differences in the body and the barriers imposed by the environment, a ramp that does not exist, a text without enlarged font, a classroom where one only learns by listening. It is in this space, in this mismatch, that disability occurs. And this completely changes the course of intervention. Because the goal ceases to be fixing the individual, mending what is prominently broken in them, and becomes the transformation of environments, the removal of barriers, the creation of conditions for full participation to occur. When we apply this logic to VR and autism, the value of the technology may lie precisely in its ability to create accessible environments, spaces where sensory stimuli are calibrated, social rules become explicit, and practice can occur without the anxiety typical of the unpredictable world [8,9].

In this sense, this article proposes exactly a theoretical reformulation of the application of VR as an environmental accessibility technology, based on the Social Model of Disability and the principles of neurodiversity. We critically analyze the available evidence, identifying documented benefits, but also important gaps regarding the safety and theoretical coherence of the studies. Then, we offer a new conceptual framework anchored in the Social Model of Disability, with concrete implications for the research, development, and implementation of these technologies. The central argument is to reposition VR as a tool for environmental accessibility, rather than an intervention focused on correcting deficits. In this way, it will be possible to formulate more relevant questions and create safe technological applications that truly meet the needs of the autistic community, in line with the principles of neurodiversity. We wish to be explicit about the nature of this contribution: this is a theoretical reformulation and perspective article, not a systematic review. We draw selectively on the literature to develop a conceptual argument, and we do not claim to provide an exhaustive or quantitative synthesis of the field.

## 2. The Dominant Paradigm: VR as Deficit Correction

If we attend any academic conference on VR and autism, we will see the same narrative repeated in numerous studies: VR trains eye contact, facial expression recognition, and joint attention. VR simulates conversations and interactions in classrooms, job interviews, and other situations to teach autistic individuals how to experience neurotypical social life [10,11,12,13,14]. Furthermore, other studies have demonstrated the effectiveness of VR in teaching safety skills [15,16], daily living skills [17,18], and emotional regulation [3]. We acknowledge that the “safety, repeatability, and predictability” inherent in VR cannot fully replicate the unpredictability of real-world neurotypical social interactions, which demand pragmatic language, executive functions, and simultaneous attentional and cognitive control at levels rarely required by other cognitive tasks. This inherent limitation reinforces, rather than invalidates, our argument that VR is most ethically and effectively employed when used to adapt environments to the individual, rather than to train them to tolerate environments that may remain sensorially and socially aversive. For individuals who choose to develop skills geared toward neurotypical contexts, the question of how virtual experiences generalize to the real world becomes an empirical priority, which we address in our research agenda. Proponents of this approach hypothesize that, given safe practice environments, autistic individuals will generalize these skills to real-world settings and overcome what are framed as their difficulties.

There are several specific examples of this approach in the literature. For example, Virtual Reality Job Interview Training (VR-JIT), which places users in front of a virtual recruiter to practice appropriate responses and nonverbal cues [10,14]. Although the goal of improving employment outcomes is legitimate, the mechanism, as typically implemented, is predominantly corrective, resting on the assumption that the autistic candidate must mask their natural communication style to be worthy of a job. Also in this format, Virtual Reality Social Cognition Training (VR-SCT) focuses on decoding complex social scenarios, often reducing human interaction to a series of logical puzzles to be solved [11]. Even Computer-Assisted Virtual Environments (CAVE) systems, which use large-scale projections instead of headsets, are often programmed with social stories that dictate exactly how an autistic child should behave in a supermarket or doctor’s office [11]. We contend that each of these applications operates within a deficit-oriented framework, treating the autistic experience as a series of errors to be corrected. We do not suggest, however, that every VR intervention for autistic people is inherently corrective, nor that skill development is objectionable in itself. Our concern is with the uncritical dominance of this framing, which we discuss further in Section 4.

However, we must be cautious when interpreting the existing evidence. According to the meta-analysis by Karami et al. [2], which analyzed 33 studies, it was shown that VR-based interventions produce moderate to large effect sizes. The strongest effect was observed in daily living skills (g = 1.15). Regarding other skills, moderate effects were observed for cognitive skills (g = 0.45), emotional regulation and recognition skills (g = 0.46), and social and communication skills (g = 0.69). More recently, Mittal et al. [3] conducted a meta-analysis and found significant improvements in social (Standardized Mean Difference (SMD) = 1.43) and emotional (SMD = 2.45) skills in children and adolescents with autism spectrum disorder (ASD). A recent systematic review by Altın et al. [19], examining 31 studies published between 2014 and 2024, corroborates these findings while again noting the heterogeneity and methodological limitations of the available evidence. However, these numbers should be interpreted with caution. Many studies were small, as they did not have active control groups and measured outcomes immediately after training, and rarely assessed generalization or long-term maintenance. The fact that acute generalization of a particular skill occurs, that is, when, for example, an autistic person learns to navigate easily through a silent virtual environment, but with the emergence of unpredictable noise or even smells and social pressures of a real environment, this skill ends up dissipating [20]. In our view, the laboratory is not the world, and a virtual avatar is not a living human being with its own biases and complexities.

Essentially, in this view, autism is treated as a skills gap, as a set of competencies that the individual does not possess and that must be acquired through repeated practice. In our reading of this literature, almost no attention is given to the sensory environment itself, such as the fluorescent lights in the classroom, the unpredictable noise in the hallway, the social pressure of an unscripted interaction. What is considered a successful intervention? Increased eye contact? Faster naming of emotions? More frequent initiation of conversations? Each of these objectives frames autistic behavior as something to be overcome, not understood. In these studies, VR becomes a tool to reinforce normative behaviors instead of a tool to transform the environment, and this represents a great opportunity to leverage technology for true inclusion.

## 3. The Unaddressed Question: Safety and Adverse Effects

Something that has been discussed in the literature, albeit still discreetly, is whether VR, instead of helping, could harm the abilities and behaviors of autistic people. The dilemma is that virtual environments are, for the most part, designed by neurotypical researchers for neurotypical people, without taking into account the sensory sensitivities that mark a large part of the autistic experience [9,21]. A classic example is so-called cybersickness, a familiar sensation of nausea, dizziness, and disorientation that arises when the visual system perceives movement, but the vestibular system insists that the body is still [22,23]. The body says one thing; the eyes say otherwise. In our view, for someone whose sensory processing already operates in a delicate balance, this mismatch can be much more than a passing phase. On this point, studies have found that cyber-nausea rates in the general population vary widely, depending on the system [22,24]. A recent systematic review by Simón-Vicente et al. [25] further confirms that cybersickness remains among the most consistently reported adverse effects of VR across applications. For autistic people, who may already exhibit atypical sensory integration, the rates may be even higher, although this has not yet been systematically tested. However, in the study we reviewed, adverse effects in autistic samples were rarely reported, and the available evidence does not yet permit field-wide conclusions [25].

Emerging evidence suggests that these risks are not merely theoretical. In their systematic review, Yang et al. [26] found that, although VR generally yielded significant improvements, adverse effects associated with its use were observed. The nature and severity of these effects varied depending on the hardware used and the participants’ sensory profiles. Among the findings, it was noted that children and adolescents with high-functioning autism (HFA) experienced dizziness and nausea when using Cave Automatic Virtual Environment (CAVE) systems, as well as anxiety and fear upon first encountering head-mounted displays (HMDs), fatigue following sessions, and headaches and eye strain, particularly after prolonged use of VR devices. Taken together, these findings underscore the urgent need for systematic safety reporting: adverse effects are neither rare nor uniform, and appear to be closely linked to individual sensory profiles and the specific characteristics of each device.

Sensory overload is another frequently underestimated challenge in the context of immersive technologies [15,26]. VR devices, for example, emit a complex range of visual and auditory stimuli, including vibrant chromaticism, rapid kinetic displacements, and multidirectional spatialized audio. A recent systematic review of immersive wearable VR in autism similarly reports cybersickness, sensory overload, and fatigue, particularly in highly immersive environments [25]. In our reading, for autistic individuals with auditory hypersensitivity, the 360-degree sound environment of an urban simulation can transcend discomfort and be processed as physically aversive [27,28]. Additionally, tactile defensiveness plays a critical role: the weight of the headset, the compression of the straps in the frontal region, and the thermal dissipation of the screen can precipitate acute states of anxiety or, in more severe scenarios, disruptive crises [29,30,31]. Another determining factor, although rarely prioritized, refers to proprioceptive difficulties, which compromise navigation and postural stabilization in virtual environments. In these cases, the incongruity between the static state of the physical body and the movement captured by the visual system generates a profoundly destabilizing vestibulo-visual conflict for a sensory processing system that already operates in a precarious balance [26].

Furthermore, it is also important to consider how individual sensory profiles affect VR tolerance [32]. Autism is not a homogeneous condition; one autistic person may seek intense visual stimuli, while another may become paralyzed by the same stimuli [33]. In the current state of affairs, VR studies tend to adopt a standardized approach for hardware and software, without offering the level of customization that would be necessary for safe application in autistic individuals [34]. The cognitive load in virtual environments is exceptionally high, where the user needs to process the simulated world, follow social scripts, and deal with the discrepancy between virtual and physical stimuli, all simultaneously [35]. One possible consequence of this sustained demand is the development of fatigue, which can further worsen the individual's condition [36,37,38]. For an autistic person who already expends extra cognitive effort during social interactions, masking can lead to rapid exhaustion and, in severe cases, burnout [39]. And the most worrying thing is that few people are paying attention to this [40].

The clinical details of this fatigue are often overlooked. This fatigue is not merely subjective tiredness; it is a complete depletion of executive function resources. According to the literature, dropout rates in VR treatment are significantly underreported; and when cited, they tend to be attributed to disinterest or lack of motivation, rather than physical, emotional, or sensory discomfort. This is a topic that needs to be discussed extensively, because if VR is implemented in autism research and practice, safety and sensory ergonomics should be treated as primary outcomes, not as an afterthought. One cannot claim that treatment is helping if the very tool being used causes sensory trauma [13].

## 4. Reframing VR: The Social Model of Disability as a Theoretical Alternative

The Social Model of Disability draws an important distinction between impairment, the biological condition (for example, autism), and disability, understood as the social and physical barriers that prevent the individual’s full participation [6,7]. In this sense, an autistic person is constrained not by their neurological condition alone, but primarily by environments built for neurotypical communication, sensory processing, and social rhythms.

By adopting the Social Model, the objective of using VR shifts from correcting or normalizing deficits to creating accessibility [6,41]. VR becomes a tool to modify the environment, building adapted sensory spaces, allowing communication to happen at the user’s pace, and simulating scenarios that, in the physical world, would be oppressive or simply unfeasible [21]. It is important to emphasize that the medical and social models are not mutually exclusive. Our critique is directed at the uncritical dominance of the deficit-correction paradigm, not at individual researchers working within the medical model, nor at the legitimate pursuit of treatments that reduce suffering and improve quality of life. Both frameworks have valuable contributions to make, and the question of what constitutes ‘cure’ remains philosophically complex. We can identify at least three explicit points of convergence. First, the question of what constitutes a ‘cure’ for autism is philosophically complex, and both models can agree that an intervention which expands communicative capacity, allowing autistic people to share their unique insights and to express their needs and preferences, is valuable in every practical and moral sense, whether or not one chooses to call it a cure. Second, both frameworks can agree that interventions which reduce suffering and improve quality of life are legitimate, provided they are chosen by the individual rather than imposed. Third, co-design benefits research within both frames: it improves the validity, safety, and acceptability of interventions regardless of whether the underlying goal is treatment or environmental adaptation, and it ensures that the priorities of the autistic community shape the questions asked in either paradigm.

This is not merely preparation for the neurotypical world, but a legitimate end in itself, which directly improves quality of life, autonomy, and freedom from sensory distress. And none of this rules out skill development chosen freely by the individual. If an autistic person decides, on their own terms, to prepare for a context they wish to inhabit, say, a job interview with a neurotypical employer, such training is entirely compatible with the social model of disability, provided it is chosen rather than imposed. This distinction, between imposed normalization and self-determined transition, is the cornerstone of our framework. A concrete example would be using VR, instead of training eye contact (which often forces behavioral masking), to create a virtual job interview where the interviewer’s avatar simply does not require eye contact, reduced lighting, or even taking pauses without missing the opportunity [10]. We recognize, however, that environmental adaptation alone is not sufficient. As one reviewer observed, most neurotypical employers will still expect eye contact, and offering autistic people only the opportunity to interact with an adapted world, without any titration toward the neurotypical world, may ultimately do them a disservice. We agree, and we do not propose that adaptation should replace preparation. Rather, we propose that preparation be self-determined rather than imposed: when an autistic person chooses, on their own terms, to develop skills for a context they wish to inhabit, VR can support that transition in a graduated and sensorially calibrated manner. This is precisely why our research agenda includes longitudinal and ecological validity studies: to examine how accessibility experiences generalize to real-world participation and how gradual transitions can be designed for those who choose them.

Seen this way, intervention ceases to be about correcting the autistic person and becomes about adapting the world and welcoming the person where they are, not where we think they should be. This reformulation is directly related to the neurodiversity paradigm, which recognizes autism as a natural variation of human neurology [42,43]. This perspective does not deny that autism can also be experienced as a biomedical condition involving genuine distress and disability; rather, it affirms that autism is simultaneously a neurological reality and an identity and that interventions should target suffering and disabling barriers without treating autistic identity itself as a pathology to be eliminated. And it is not just a theoretical issue, as the self-advocacy movement within the autistic communities themselves has taken a clear stance on technology. Often, the criticisms are harsh, especially against solutions that function more like digital leashes than support tools [44]. And what they defend, rightfully so, are technologies that expand autonomy and self-determination [45], technologies that do not teach the person to fit in, but that expand what they can do based on who they are. 

How does this translate into practice? In practice, through participatory design. Instead of researchers deciding that an autistic person needs to learn to small talk, participatory design involves autistic individuals from day one [44]. They might discover that what they really need is a VR simulation of a crowded airport to help them identify quiet zones, or a virtual social space where they can interact through text or gestures, rather than forced speech [45,46]. Recent work reinforces that involving autistic people as partners in the design process, rather than as passive participants, is both feasible and valuable, including with autistic adults [46].

In this model, the success of a VR application is measured by how much it reduces the disabling barriers of the environment. It is not that this perspective is entirely absent from recent literature, but it is still under-discussed. A substantial portion of VR studies in autism remains within the deficit paradigm, although notable exceptions exist and are growing [44,46]. The Social Model offers a coherent alternative, which requires us to ask not what is wrong with this person, but what is wrong with this environment and how VR can help change it [6,42]. In our view, it is the difference between teaching someone to endure a sensorially aversive and dysregulatory environment and providing opportunities for its restructuring.

## 5. Future Directions and Clinical Implications

Moving from theory to practice requires more than just good intentions. Therefore, this section presents four research priorities that embody this reformulation, with the first undoubtedly being the most urgent.

Systematic documentation of adverse effects needs to become the standard, not the exception [20]. Every VR study with autistic participants should, at a minimum, report rates of cybersickness, sensory discomfort, anxiety levels, and fatigue, preferably using validated instruments and, ideally, adapted for autistic populations [13,46]. We need to establish safety limits that take sensory hypersensitivity into account, not as a secondary variable, but as a central factor in the design [4]. Without this data, we simply cannot claim that VR is safe. And without this guarantee, we risk causing harm precisely when we think we are helping.

Participatory co-design must also include minimally verbal and non-speaking autistic individuals, a significant subpopulation frequently excluded from participatory research. Virtual tools are particularly well suited to this task: these individuals can contribute through choices expressed via behaviors distinct from speech and language, including gestures, eye gaze, physiological responses, and behavioral preferences, and VR environments can be designed to elicit and capture such non-verbal preference signals in ways that traditional co-design settings may not [46].

Secondly, participatory design is not an extra; it is a condition for operation. Future research needs to involve autistic people as co-designers at every stage of the process, from the initial concept to the final implementation [44]. And here we are not talking about symbolic advisory boards that exist more for appearances than for substantive decision-making. We are talking about real collaboration, where the autistic people themselves define the research questions and what success means [46]. If the intention is for VR to serve the autistic community, the community needs to have an active voice in the design process, not as validators of something already finished, but as protagonists. Only then will the software reflect lived experience, and not the outsider’s perspective, the distant observation of a researcher who has never experienced what they are trying to measure [43].

The third point concerns metrics. When considering the Social Model, outcome measures also need to change [6]. Instead of monitoring whether an autistic person is making more eye contact after training, we should develop accessibility metrics. For example, how autonomous the autistic person feels, whether they can be present and interact in a space without sensory distress, and how well the virtual environment can be adapted to their sensory profile [21]. In this sense, it should be verified not whether this person has learned to behave neurotypically, but whether VR has reduced or increased the cognitive load of this autistic person’s daily life. Within this context, success should be defined by considering quality of life and functional autonomy, and not by the accuracy with which this autistic person can imitate a behavior that is not natural to them [42].

Fourth, longitudinal and ecological validity studies are still lacking [2]. Most VR and autism studies measure immediate effects in the laboratory. These studies should also examine gradual transitions for individuals who choose to engage with neurotypical contexts, and how VR-based accessibility experiences generalize to real-world participation. Regarding the benefits observed in this study, it is necessary to check if they translate to the real world and if they are also lasting [3]. Long-term study, with follow-up periods of 6 to 12 months, are needed to check if VR as an accessibility tool provides sustained improvements in community participation [46]. Furthermore, it is also important to understand the long-term effects of repeated exposure to immersive virtual environments, both positive and negative. VR is still a relatively new form of treatment, and there is still insufficient data on its effects on autism. Now is the time to conduct a serious analysis of the impact of VR on identity and well-being, and not let enthusiasm distract us from the right path [45].

## 6. Conclusions

VR holds genuine promise for autistic people, and the positive findings reviewed here are not in dispute. What we question is not the technology itself, but the dominant framework within which it is often deployed. As the evidence we have reviewed suggests, much of this research has framed VR as a corrective tool, aimed at social or emotional differences that researchers have identified as deficits. We argue that this framing is difficult to defend, both theoretically and ethically, and that it risks obscuring the safety concerns and the limited involvement of autistic people in the design of these tools. Side effects have not been properly studied, and autistic people rarely have an active voice in the development of these tools. We need to look beyond the screen, and beyond the exciting results surrounding the technology, at the real person using the headset. The initial findings of VR studies deserve attention, and we do not dispute them. What we question is the interpretive lens through which they are often read. We argue that they need to be interpreted from a perspective that takes safety seriously and respects neurodivergent ways of experiencing the world. This requires more than the kind of respect mentioned in the limitations section of an article; it demands a respect that shapes how these technologies are built from the very beginning. When this perspective is considered within the framework of the Social Model of Disability and Neurodiversity, the landscape shifts. We stop seeing VR as a tool for correcting autistic behaviors and begin to understand it as a technology that should adapt to the person, rather than the other way around. In our view, this perspective moves us away from focusing exclusively on curing autism and toward designing an accessible world where autistic people can participate fully, without the pressure of hiding who they are, while remaining open to interventions that alleviate suffering and expand communicative capacity for those who seek them. We therefore prioritize safety and real-life experience, focusing on the individual’s well-being rather than on how well they can imitate neurotypical behavior. Adopting this stance, we believe, allows VR to become a valuable and liberating resource in the lives of autistic people, rather than just another instrument with a corrective purpose. In our view, the screen is not the limit; what limits us is our collective imagination and our willingness to listen.
